# Short-term streamflow modeling using data-intelligence evolutionary machine learning models

**DOI:** 10.1038/s41598-023-41113-5

**Published:** 2023-08-24

**Authors:** Alfeu D. Martinho, Henrique S. Hippert, Leonardo Goliatt

**Affiliations:** 1 Exact Sciences and Technology Department, Púnguè University, Tete Delegation, Campus Universitário de Cambinde-EN106, Matundo, Tete Mozambique; 2 Statistics Department, Federal University of Juiz de Fora, Campus Universitário, Rua José Lourenço Kelmer, s/n-São Pedro, Juiz de Fora, Minas Gerais Brazil; 3 Computational and Applied Mechanics Department, Federal University of Juiz de Fora, Campus Universitário, Rua José Lourenço Kelmer, s/n–São Pedro, Juiz de Fora, Minas Gerais Brazil

**Keywords:** Environmental sciences, Hydrology

## Abstract

Accurate streamflow prediction is essential for efficient water resources management. Machine learning (ML) models are the tools to meet this need. This paper presents a comparative research study focusing on hybridizing ML models with bioinspired optimization algorithms (BOA) for short-term multistep streamflow forecasting. Specifically, we focus on applying XGB, MARS, ELM, EN, and SVR models and various BOA, including PSO, GA, and DE, for selecting model parameters. The performances of the resulting hybrid models are compared using performance statistics, graphical analysis, and hypothesis testing. The results show that the hybridization of BOA with ML models demonstrates significant potential as a data-driven approach for short-term multistep streamflow forecasting. The PSO algorithm proved superior to the DE and GA algorithms in determining the optimal hyperparameters of ML models for each step of the considered time horizon. When applied with all BOA, the XGB model outperformed the others (SVR, MARS, ELM, and EN), best predicting the different steps ahead. XGB integrated with PSO emerged as the superior model, according to the considered performance measures and the results of the statistical tests. The proposed XGB hybrid model is a superior alternative to the current daily flow forecast, crucial for water resources planning and management.

## Introduction

Given the scarcity of water and the concerns about its future availability, it is essential to undertake studies that can aid in comprehending its dynamics for effective management. However, the variability of this water resource, attributed to climate change phenomena such as severe droughts, floods, storms, cyclones, and even human actions^1^ exhibits chaotic, non-linear characteristics and high stochasticity^2^, making prediction complex and still a significant challenge.

Machine learning models are currently used as alternatives to deal with this complexity; however, they often underperform due to their dependence on the chosen parameters. Various evolutionary search algorithms, such as genetic algorithms (GA), firefly algorithm (FFA), particle swarm optimization (PSO), salp swarm algorithm (SSA), gray wolf optimization (GWO), spotted hyena optimizer (SHO), differential evolution (DE), cuckoo search algorithm (CSA), Ant colony optimization (ACO), and even multi-objective optimization design (MOOD), have been proposed. These algorithms have demonstrated excellent global optimum search capabilities compared to classic optimization methods, leading to the development of hybrid models^3–15^. The application of these hybrid approaches for predicting hydrological variables is a relatively new technique that has shown significant improvement in forecasting^16–24^. Recent studies along these lines have been conducted for predicting river flows^25–31^.

The multivariate adaptive regression spline (MARS) model, combined with the differential evolution (MARS-DE) algorithm, was developed by^32^ to simulate water flow in a semi-arid environment, using antecedent values as inputs. According to the authors, the MARS-DE model demonstrated strong hybrid predictive modeling capabilities for water flow on a monthly timescale compared to LSSVR and the standard MARS model.

Multi-objective optimization design (MOOD) was employed by^33^ to select and fine-tune the weights of the models extreme learning machine (ELM) and echo state network (ESN), resulting in the hybrid models ELM-MOB and ESN-MOB. These hybrid models were developed for influent flow forecasting using past values as input variables. The results of these models were compared with the SARIMA model, demonstrating their superior performance. Specifically, the ESN-MOB model outperformed the others.

The prediction accuracy of the ANFIS-FFA hybrid model, which combines adaptive neuro-fuzzy inference systems (ANFIS) and the firefly algorithm (FFA), was evaluated by^34^ in predicting throughput using their antecedent values as inputs. The proposed hybrid model was compared with the classical version (ANFIS). The outcomes revealed that the FFA could enhance the prediction precision of the ANFIS hybrid model.

A study conducted by^35^ obtained results similar to those of^34^ when combining ANFIS and PSO (ANFIS-PSO). The proposed hybrid approach demonstrated the ability to generate accurate estimates for modeling upstream and downstream daily flows, in comparison to other approaches such as MARS and M5tree. Precipitation and discharge were used as input data for the model. In another study, Yaseen et al.^36^ developed a hybrid model named the extreme learning machine model (ELM) with the salp swarm algorithm (SSA-ELM). The developed model was compared with the classic ELM and other artificial intelligence (AI) models in monthly flow forecasting, utilizing antecedent values as inputs. The flow prediction precision of SSA-ELM exceeded that of the classic ELM and other AI models.

A recent algorithm called gray wolf optimization (GWO) was applied to enhance the effectiveness of artificial intelligence (AI) models by^37^. The findings indicated that AI models with integrated GWO (ANN-GWO, SVR-GWO, and MLR-GWO) outperformed standard AI methods such as ANN and SVR. Additionally, SVR-GWO exhibited better performance in predicting monthly flow compared to ANN-GWO and MLR-GWO. In another study, Tikhamarine et al.^38^, applied GWO in combination with Wavelet SVR (GWO-WSVR). The results showed that the GWO algorithm outperformed other optimization approaches like Particle swarm optimization (PSO-WSVR), shuffled complex evolution (SCE-WSVR), and multi-verse optimization (MVO-WSVR). These methods were also employed in tuning WSVR parameters, revealing the superiority of GWO in optimizing standard SVR parameters to improve flow prediction accuracy. Both studies used only past flow values as input variables.

The prediction capability of support vector regression (SVR) was optimized using various algorithms, namely spotted Hyena optimizer (SVR-SHO), ant lion optimization (SVR-ALO), Bayesian optimization (SVR-BO), multi-verse optimizer (SVR-MVO), Harris Hawks optimization (SVR-HHO), and particle swarm optimization (SVR-PSO). These algorithms were used to select the SVR parameters and were tested by^39^. The comparison results showed that SVR-HHO outperformed the SVR-SHO, SVR-ALO, SVR-BO, SVR-MVO, and SVR-PSO models in daily flow forecasting in the study basin, utilizing past flow values as input variables. In comparison with the competition, the new HHO algorithm demonstrated superior performance in making predictions.

The performance of extreme learning machine (ELM) models optimized by bioinspired algorithms, namely ELM with ant colony optimization (ELM-ACO), ELM with genetic algorithm (ELM-GA), ELM with flower pollination algorithm (ELM-FPA), and ELM with Cuckoo search algorithm (ELM-CSA), was compared in a study by^40^, for the prediction of evapotranspiration (ETo). The proposed models were evaluated and contrasted with the standard ELM model. The results indicated a greater ability of the bioinspired optimization algorithms to enhance the performance of the traditional ELM model in daily ETo prediction, particularly the FPA and CSA algorithms.

A new hybrid model for monthly flow forecasting, named ELM-PSOGWO (integrating PSO and GWO with ELM), was proposed by^41^. This approach was compared with the standard ELM, ELM-PSO, and ELM-PSOGSA methods (hybrid ELM with integrated PSO and binary gravitational search algorithm). The models were tested for accuracy using monthly precipitation and discharge data as inputs. The results indicated that the ELM-PSOGWO model outperformed the competition, demonstrating the ability to provide more reliable predictions of peak flows with the lowest mean absolute relative error compared to other techniques.

The deep learning hybrid model, known as the gray wolf algorithm (GWO)-based recurrent gated unit (GRU) (GWO-GRU), was developed by^42^ for forecasting daily flow rates, utilizing its antecedents as input variables. The proposed model was compared with a linear model. According to the findings, GWO-GRU outperforms the linear model.

The performance of the support vector machine hybrid model with particle swarm optimization (PSO-SVM) was evaluated by^43^ for short-term daily flow forecasting in rivers. The model used river flow, precipitation, evaporation, average relative humidity, flow velocity, average wind speed, and maximum and minimum temperature as input variables. The outcomes demonstrated that the hybrid model outperformed the standard SVM in predicting flow 1–7 days ahead. Furthermore, they found that the inclusion of meteorological variables improved flow prediction.

A hybrid model based on the integration of hybrid particle swarm optimization and gravitational search algorithms (PSOGSA) into a feed-forward neural network (FNN) (PSOGSA-FNN) was developed by^44^ for forecasting monthly flow, using its antecedent values as predictors. The outcomes indicated that the proposed model achieved better forecast accuracy and is a viable method for predicting river flow.

Various evolutionary algorithms, such as genetic algorithm (GA), fire-fly algorithm (FFA), gray wolf optimization (GWO), differential evolution (DE), and particle swarm optimization (PSO), were coupled with ANFIS and trained and tested for forecasting daily, weekly, monthly, and annual runoff using runoff antecedents as inputs by^45^. The findings showed that the hybrid algorithms significantly outperformed the conventional ANFIS model for all forecast horizons. Furthermore, ANFIS-GWO was identified as the superior hybrid model. In another study by^46^, a hybrid ANFIS model with integrated gradient-based optimization (GBO) was proposed for flow forecasting, using temperature data and antecedent flow values as predictors. The outcomes revealed that the proposed model is superior to the standard ANFIS.

In the same perspective, Haznedar and Kilinc^29^ developed a hybrid ANFIS model with an integrated genetic algorithm (GA) (ANFIS-GA) for streamflow prediction, using its past values as input. The outcomes demonstrated that the suggested model performs better than the standard ANFIS, LSTM, and ANN. Dehghani et al.^47^ applied the GWO-optimized ANFIS model for the recursive multi-step forecast of flow between 5 min and 10 days ahead, using antecedent values as inputs, and observed that the proposed model outperformed the standard in all forecast horizons.

Hybrid machine learning models were tested for flood prediction by^19^. In their study, the authors applied GWO-optimized MLP and SVR models (MLP-GWO and SVR-GWO) and observed that SVR-GWO achieved superior results compared to MLP-GWO. The results also demonstrate that using GWO as an optimizer results in a potential improvement in the performance of MLP and SVM models for flood forecasting.

Other recent hybrid approaches aimed at enhancing the performance of machine learning (ML) models for streamflow forecasting also deserve special mention. These include the linear and stratified selection in deep learning algorithms by^48^; forest-based algorithms applied to neural network models as investigated by^49^; the use of meta-heuristic algorithms (MHA) in artificial neural networks (ANN) as explored by^50^; PSO integration for parameter selection in ANN^51^; and novel hybrid approaches based on conceptual and data-driven techniques^52^. Table 1 presents the summary of some hybrid models resulting from the optimization of the parameters.

**Table 1:** Summary of hybrid artificial intelligence methods by parameter optimization for flow forecasting.

Reference	Case study	Hybrid model
^32^	Iraq	Differential evolution integrated into multivariate adaptive regression spline (MARS-DE)
^33^	Brazil	Echo state network and multi-objective optimization design (ESN-MOB)
^34^	Malaysia	Firefly optimization algorithm and adaptive neuro-fuzzy inference systems (ANFIS-FFA)
^35^	Pakistan	Particle swarm optimization algorithm and neuro-fuzzy inference systems (ANFIS-PSO)
^37^	Egypt	support vector regression with grey wolf optimization (GWO-SVR)
^38^	Algeria	Wavelet support vector regression with grey wolf optimization (GWO-WSVR)
^39^	India	Harris Hawks optimization and support vector regression (SVR-HHO)
^40^	China	Extreme learning machine with flower pollination algorithm (ELM-FPA)
^41^	Pakistan	Particle swarm optimization and gray wolf optimization with extreme learning machine (ELM-PSOGWO)
^53^	Iran	Support vector regression optimized by grasshopper optimization algorithm (GOA) with LASSO input selection
^36^	Iraq	Extreme learning machine model with salp swarm algorithm (SSA-ELM)
^42^	Turkey	Gated recurrent unit with grey wolf algorithm (GWO-GRU)
^43^	Malaysia	Particle swarm optimization and support vector machine (PSO-SVM)
^44^	Tukey	Hybrid particle swarm optimization and gravitational search algorithms with feed-forward neural network (FFN-PSOGSA)
^45^	Iran	Adaptive neuro-fuzzy inference systems with grey wolf optimization algorithm (GWO-ANFIS)
^46^	Pakistan	ANFIS with gradient-based optimization (GBO) (GBO-ANFIS)
^47^	Iran	ANFIS with GWO

As noted earlier, many studies compare hybrid models with their corresponding standard models or compare the same ML model using different algorithms to select their parameters, often applied for one-step-ahead streamflow forecasting. In this work, various ML models are combined with different optimization algorithms for parameter selection, allowing us to identify not only the best ML model and the optimal parameter optimization algorithm but also the best hybrid model among those developed. It’s also important to highlight its application to multi-step-ahead forecasting, an area that is still relatively unexplored in the literature. From another perspective, there are very few studies on the combination of ML and hydrology in Africa, particularly in Mozambique. This work aims to address this gap, which represents a novel contribution to the field.

This study compares the performance of machine learning models combined with the genetic algorithm (GA), differential evolution (DE), and particle swarm optimization (PSO) algorithms for modeling and forecasting the flow of the Zambezi River, which is a tributary to the Cahora-Bassa hydroelectric dam in Mozambique. The forecasts are conducted within a short-term time horizon, specifically considering forecast horizons of 1, 3, 5, and 7 days ahead (a multistep-ahead forecasting strategy).

The paper is organized as follows: “Materials and methods” covers the study area, data, machine learning models, bioinspired optimization algorithms applied, and the proposed methodology. “Result and discussion” presents the results of the computational experiments, along with comparative analysis and discussion. Finally, “Conclusion” provides the conclusion.

## Materials and methods

This section outlines the materials and methods employed in this study. It encompasses the study area and data, the machine learning (ML) models, and bioinspired algorithms utilized, and concludes with an overview of the proposed methodology.

### Study area and data

**Figure 1:**
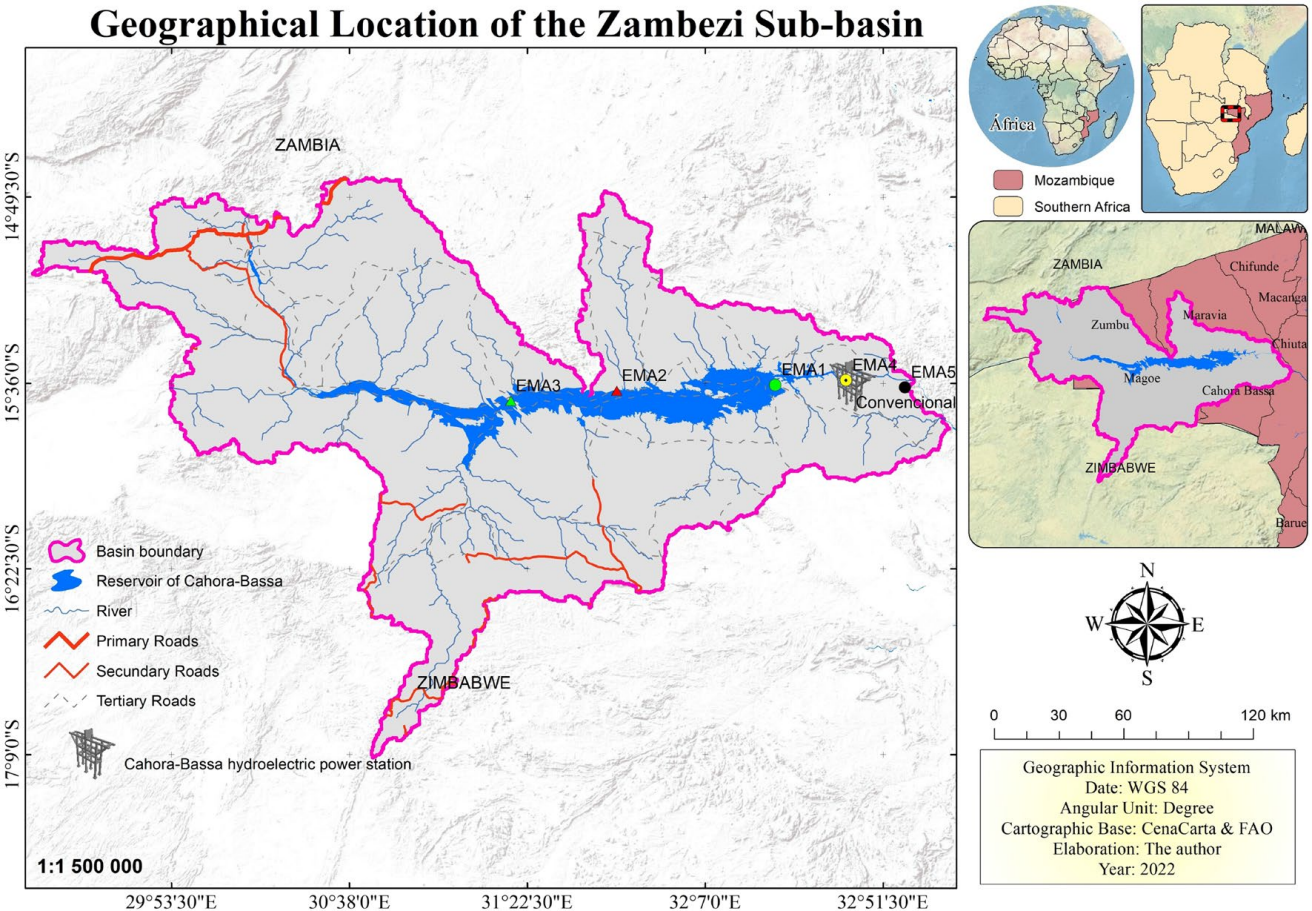
Location of the study area. The EMAs points indicate the automatic monitoring stations where the data under analysis in this work are collected^54^.

The research area is situated in a sub-basin of the Zambezi River, specifically, the Medium Zambezi terminal, located upstream of the Cahora Bassa dam in Tete province, Mozambique. Figure 1 depicts the automatic monitoring stations used in this study.

The Cahora-Bassa dam plays a critical role in Mozambique as it supplies the majority of the country’s electricity and that of neighboring regions. Additionally, it supports downstream economic activities in the Zambezi River delta, such as farming, pastoralist work, fishing, and the construction of access roads. The dam also contributes to mitigating natural disasters like droughts and floods.

Daily flow forecasts are indispensable for the operation of hydroelectric plants, including tasks such as optimizing the dam’s storage capacity, operational procedures, energy generation management, maintenance of ecological flows in the reservoir, and obtaining continuous flow records in non-calibrated catchments where direct measurements are unavailable.

The historical data analyzed in this research was provided by the Department of Water Resources and Environment of Hidroeléctrica de Cahora-Bassa (HCB), the largest electricity producer in Mozambique and the entity managing the Cahora-Bassa dam.

**Figure 2:**
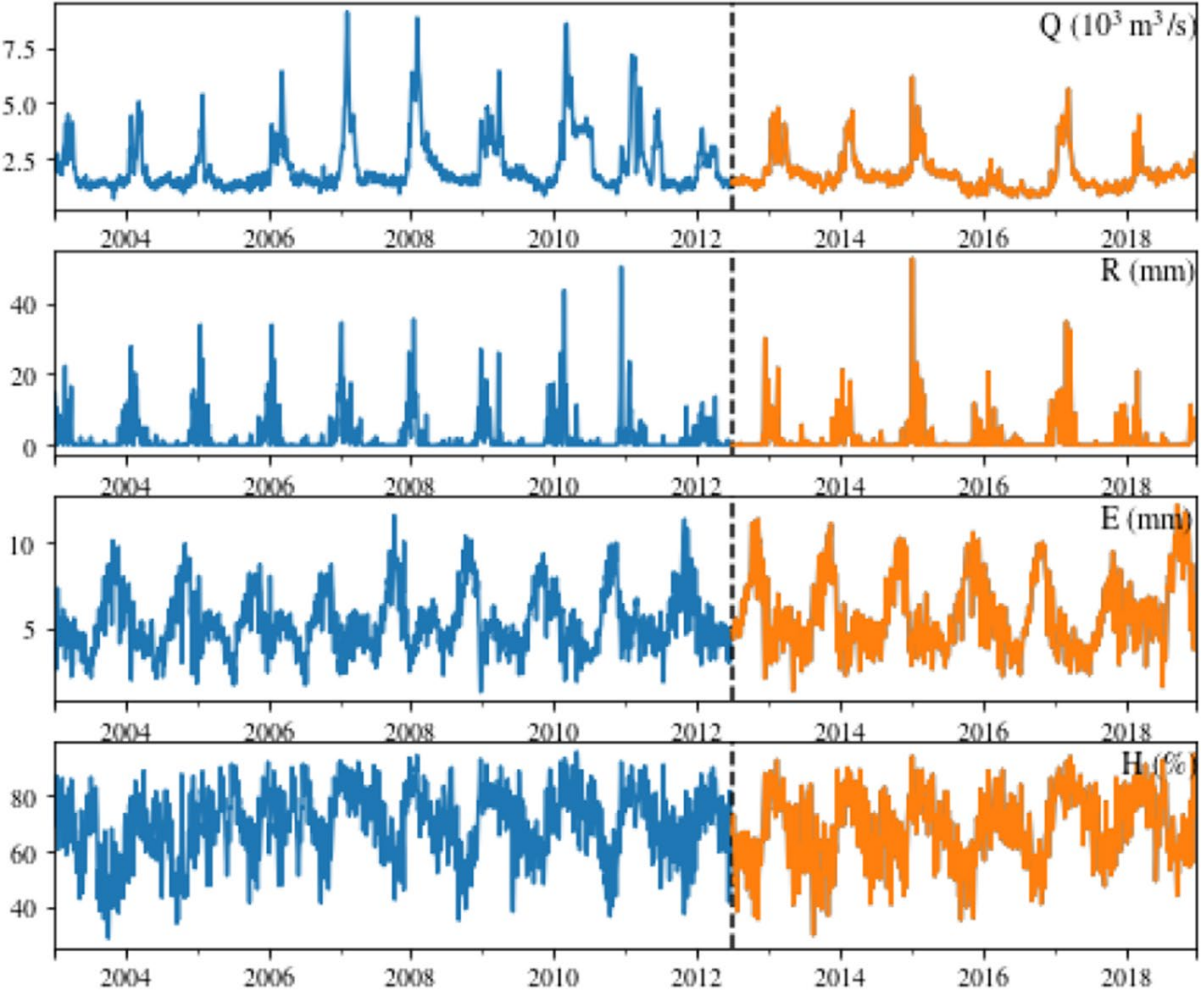
Daily data, total: 5844, between 2003 and 2018 (15 years). From 01/01/2003 to 06/30/2012 training (blue) and 07/01/2012 to 12/31/2018 test (orange)^54^.

The dataset comprises daily time series for variables, including affluent flows (Q), precipitation (R), evaporation (E), and relative humidity (H). The dataset consists of 5844 observations, spanning from 2003 to 2018, divided into two subsets: the training and testing sets. It’s important to emphasize the seasonal characteristics of these variables. Figure 2 illustrates the training set in blue, ranging from 01/01/2003 to 06/30/2012, and the test set in orange, covering the period from 07/01/2012 to 12/31/2018.

It’s worth noting that the data analyzed in this study has been used in previous research conducted by the same authors^26,54–57^.

### Machine learning models

This section provides a brief description of the machine learning (ML) models utilized in this study, which include extreme gradient boosting, elastic net, multivariate adaptive regression spline, extreme learning machine, and support vector regression.

#### Extreme gradient boosting (XGB)

XGB^58–61^ is an ensemble method that combines weak predictors to generate a strong predictor. XGB prediction for an *i* instance is

$$\begin{aligned} \hat{y_i} =\psi (x_i) =\sum _{r= 1}^ {R}g_r(x_i), ~g_r \in G \end{aligned}$$

where $$G={g(x)=w_{q(x)}} (q: R^{m} \rightarrow T, w \in R^{T})$$.

#### Support vector regression (SVR)

SVR^17,62–64^ is a classic regression method that estimation function is:

$$\begin{aligned} g(\textbf{x})=({w}.\psi (\textbf{x}))+b \end{aligned}$$

where $\psi (\textbf{x})$ is a kernel function in a feature space, $\textbf{w}$ is the weight vector, *b* is a bias, and *N* is the number of samples.

#### Elastic net (EN)

EN^65–68^ is the generalized linear model expressed as:

1 $$\begin{aligned} \min _{w} { \frac{1}{2N} \Vert \textbf{X} \textbf{w} - \textbf{y}\Vert _2 ^ 2 + \frac{\alpha \rho }{2}( 2\Vert \textbf{w}\Vert _1 - \Vert \textbf{w}\Vert _2 ^ 2)+ \frac{\alpha }{2} \Vert \textbf{w}\Vert _2 ^ 2 } \end{aligned}$$

where $\alpha \ge 0$, $\Vert \textbf{w}\Vert _2$ and $\Vert \textbf{w}\Vert _2$ are respectively the norm $L_1$ and the norm $L_2$ of the parameter array, and $\rho$ is the the parameter’s rate $L_1$.

#### Multivariate adaptive regression spline (MARS)

MARS^69–71^ is the method consist of sequential piecewise linear regression splines of the form:

2 $$\begin{aligned} \hat{y}(x) = F_m(x) = c_0 + \sum _{m=1}^{M} c_m B^K_m(x), \quad B^K_m(x) = \prod _{k=1}^{K} [\pm (x-s)]^{r}_{+} \end{aligned}$$

where $c_0$ is a constant quantity, $B^ K_m(x)$ the *m*-th basis function, and $c_m$ is the unknown coefficient.

#### Extreme learning machine

ELM^72–74^ is an artificial neural network described by Eq. (3), i.e there are ${\beta }_{i}$, $\textbf{w}_{i}$ and $b_{i}$ such that:

3 $$\begin{aligned} \sum _{i=1}^{L} {\beta }_{i} g\left( \textbf{w}_{i} \cdot \textbf{x}_{j}+b_{i}\right) =\textbf{t}_{j}, \quad j=1, \ldots , N \end{aligned}$$

where $\textbf{w}_{i}$ is the *i*-th neuron in the hidden layer, $\beta _{i}$ is the connection weight of the *i*th neuron of the hidden layer and the neuron of the output layer, $b_i$ is the bias of the *i*th neuron of the hidden layer, and $g(\cdot )$ denotes an activation function.

### Bioinspired optimization algorithms

Reservoir operation optimization is a complex nonlinear problem, involving a large number of decision variables and multiple constraints. In the field of water resources, various metaheuristic algorithms have been employed to address this issue. These methods often involve modifying existing algorithms or creating hybrid algorithms, ultimately contributing to the reduction of water deficits in reservoirs^75^. In this study, we employ and integrate three algorithms, which are described below. These algorithms play a crucial role in our machine learning (ML) models, aiding in the selection of optimal parameters.

#### Genetic algorithm (GA)

GA is a subclass of evolutionary algorithms used for the objective of optimization via natural genetics and selection^76^. The genetic operations are crossover, reproduction, and mutation^77^.

In the GA, a set of potential solutions to a problem are generated randomly. Each solution is evaluated using the adequacy function, which is intended to be optimized. New solutions is generated probabilistically from the best ones of the previous step, and some of these are inserted directly into the new population, while others are used as a basis to generate new individuals, using genetic operators^76,78^.

#### Diferential evolution (DE)

DE^32,79,80^ is a nature-inspired algorithm that adapts the individuals through mutation genetic operators, recombination, and selection^81^. DE consists of the following^82^ steps:

1. Initialization of parameters
2. Population initialization.
  4 $$\begin{aligned} X_{i,j}=rand_{i,j}[0,1](X_{j,max}-X_{j,min})+X_{j,min} \end{aligned}$$
3. Population evaluation: Compute and note each individual’s fitness scores.
4. Mutation operation:
  5 $$\begin{aligned} X^{\prime }_{a}=X_{a}+F(X_{b}-X_{c}) \end{aligned}$$
5. Crossover operation, according to the equation:
  6 $$\begin{aligned} \left\{ \begin{array}{ll} X^{\prime }_{b}(j)=X^{\prime }_{a}(j) &{} \text {if} ~C \ge rand(j) ~ \text {or}~ j= randn(j) \\ X^{\prime }_{b}(j)=X_{a}(j) &{} \text {Other cases} \end{array} \right. \end{aligned}$$
6. Selection operation, according to the equation:
  7 $$\begin{aligned} T_{i,G+1}=\left\{ \begin{array}{ll} T_{i,G} &{} \text {if} ~g(X_{i,G}) \ge g(T_{i,G}) \\ X_{i,G} &{} \text {Other cases} \end{array} \right. \end{aligned}$$

#### Particle swarm optimization (PSO)

PSO^83^ is an algorithm based on the natural movements of biological swarms (flocks of birds) considering their position and speed^41^.

The PSO formula for the initial iteration is:

8 $$\begin{aligned} \left\{ \begin{array}{ll} P_{i+1}=P_i+V_{i+1}\\ V_{i+1}= aV_i+c_{1}r_{1}(P_i-P_b)+c_{2}r_{2}(P_i-P_g) \end{array} \right. \end{aligned}$$

where $P_i$ and $V_i$ are, respectively, the particle’s position and speed, $P_g$ best position in the swarm and $P_b$ best personal value

### Proposed methodology

A set of real data comprising four time series was used in the analysis: the river’s flow into the reservoir for electricity generation (*Q*), precipitation (*R*), evaporation (*E*), and humidity (*H*). The affluent flow serves as the output variable, while the rest are employed as input or predictor variables.

The task of forecasting several steps ahead in the inflow was initially approached by constructing a framework that includes input variables and their corresponding lags or delays, to accommodate the proposed machine learning models.

The determination of the number of lags/antecedents or delays for making predictions of the river’s affluent flow $Q_{t+j}$ in the time horizon ($j=1, 3, 5, 7$) was accomplished using partial autocorrelation functions (PACF), autocorrelation functions (ACF), and cross-correlation function (CCF). These methods serve as a straightforward means to suggest the number of antecedents, aiding in identifying the factors influencing the output variable.

**Figure 3:**
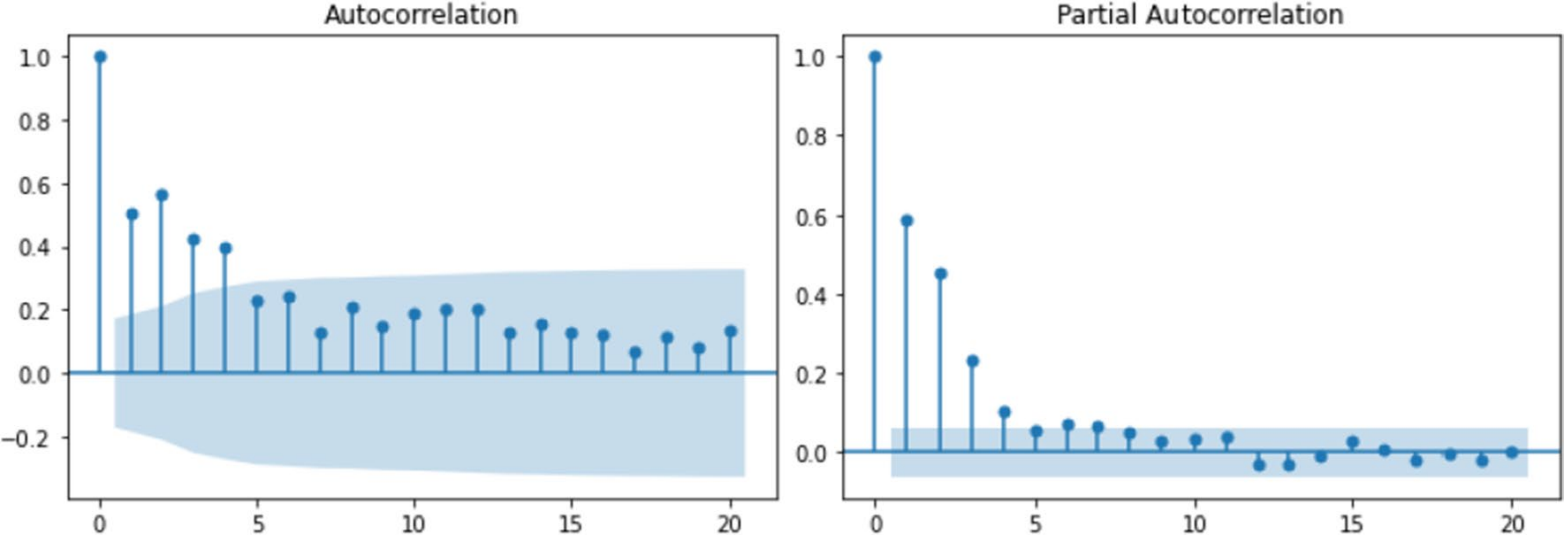
Autocorrelation and partial autocorrelation functions. Lags within the shaded part are considered statistically non-significant^54^.

**Figure 4:**
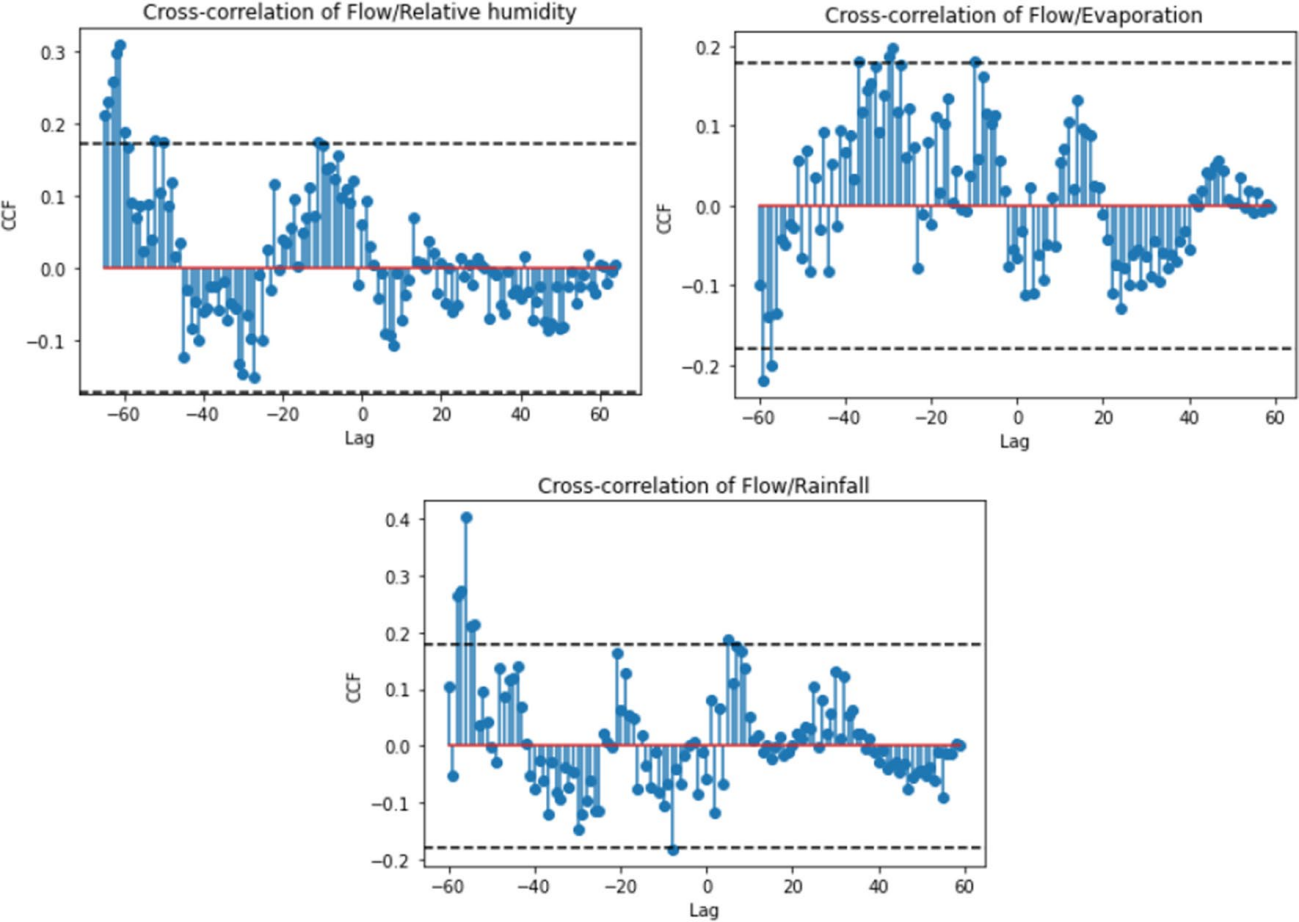
Cross correlation functions between flow and precipitation, evaporation or relative humidity. The lags between the dashed lines are considered statistically non-significant^54^.

Autoregression analysis using ACF/PACF and CCF for the analyzed variables is depicted in Figs. 3 and 4, respectively. Figure 3 suggests that early lags may be predictive, while Fig. 4 indicates that none or all lags could potentially be used as a CCF selection criterion. Furthermore, in Fig. 4, a cyclical pattern (seasonality) is noticeable, identified by the decline of correlation in certain time intervals (days).

In this study, seven lags, corresponding to twenty-eight (28) input variables (4 original variables $\times$ 7 lags), i.e., a $5844 \times 28$ matrix, were considered as input data for machine learning models using ACF/PACF as the selection criterion, since CCF was uninformative, given that the majority of lags fall within the dashed-line confidence interval, as shown in Fig. 4.

The non-significant autocorrelation observed between the response variable and the predictor variables in Fig. 4 may be attributed to the use of ACF/PACF or CCF, which are linear models. As a result, they may not detect hidden non-linear relationships or the frequent spatial variation in hydrological variables, as data from only one region were considered. Additionally, these models do not incorporate equations or physical relationships between flow and other variables. The presence of noise and hydrometeorological differences between training and test periods, known as non-stationarity, can also weaken this relationship^84,85^.

The machine learning models used in this study were elastic net (EN), extreme learning machine (ELM), extreme gradient boosting (XGB), support vector regression (SVR), and multivariate adaptive regression spline (MARS). The primary objective was to evaluate their predictive capabilities for flow across different time horizons.

These models had their parameters intelligently determined using bioinspired algorithms: differential evolution (DE), genetic algorithms (GA), and particle swarm optimization (PSO). The optimization problem’s objective function was the minimization of root mean square error (RMSE) calculated on the training set through a 5-fold walk-forward approach^86^. The experiments were conducted a total of 30 times, employing different random seeds. Table 2 illustrates the encoding of candidate solutions for each machine learning model to be used in each bioinspired algorithm.

**Table 2:** Candidate solutions’ coding.

Estimator	IP	Description	Settings/range
EN	$\theta _{1}$	Penalty term, $\alpha$	[$10^{-6}, 2$]
	$\theta _{2}$	$L_1$-ratio parameter, $\rho$	[0,1]
ELM	$\theta _{1}$	No. neurons in the hidden layer, *L*	[1, 500]
	$\theta _{2}$	Regularization parameter *C*	[0.0001, 10000]
	$\theta _{3}$	Activation function *G*	1: Identity; 2: Sigmoid; 3: Hyperbolic Tangent; 4: Gaussian; 5: Swish; 6: ReLU;
SVR	$\theta _{1}$	Loss parameter, $\varepsilon$	[10$^{-5}$, 100]
	$\theta _{2}$	Regularization parameter, *C*	[1, 10000]
	$\theta _{3}$	Bandwidth parameter, $\gamma$	[0.001, 10]
MARS	$\theta _{1}$	Degree of piecewise polynomials, *q*	[0,3]
	$\theta _{2}$	Penalty factor, $\gamma$	[1, 9]
	$\theta _{3}$	Maximum number of terms, *M*	[1, 500]
XGB	$\theta _{1}$	Learning rate, $\eta$	[10$^{-6}$, 1]
	$\theta _{2}$	No. weak estimators, $M_{est}$	[10, 500]
	$\theta _{3}$	Maximum depth, $m_{depth}$	[1, 20]
	$\theta _{4}$	Regularization parameter, $\lambda _{reg}$	[0, 100]

The IP column denotes the internal parameter used in the bioinspired algorithms’ encoding.

**Figure 5:**
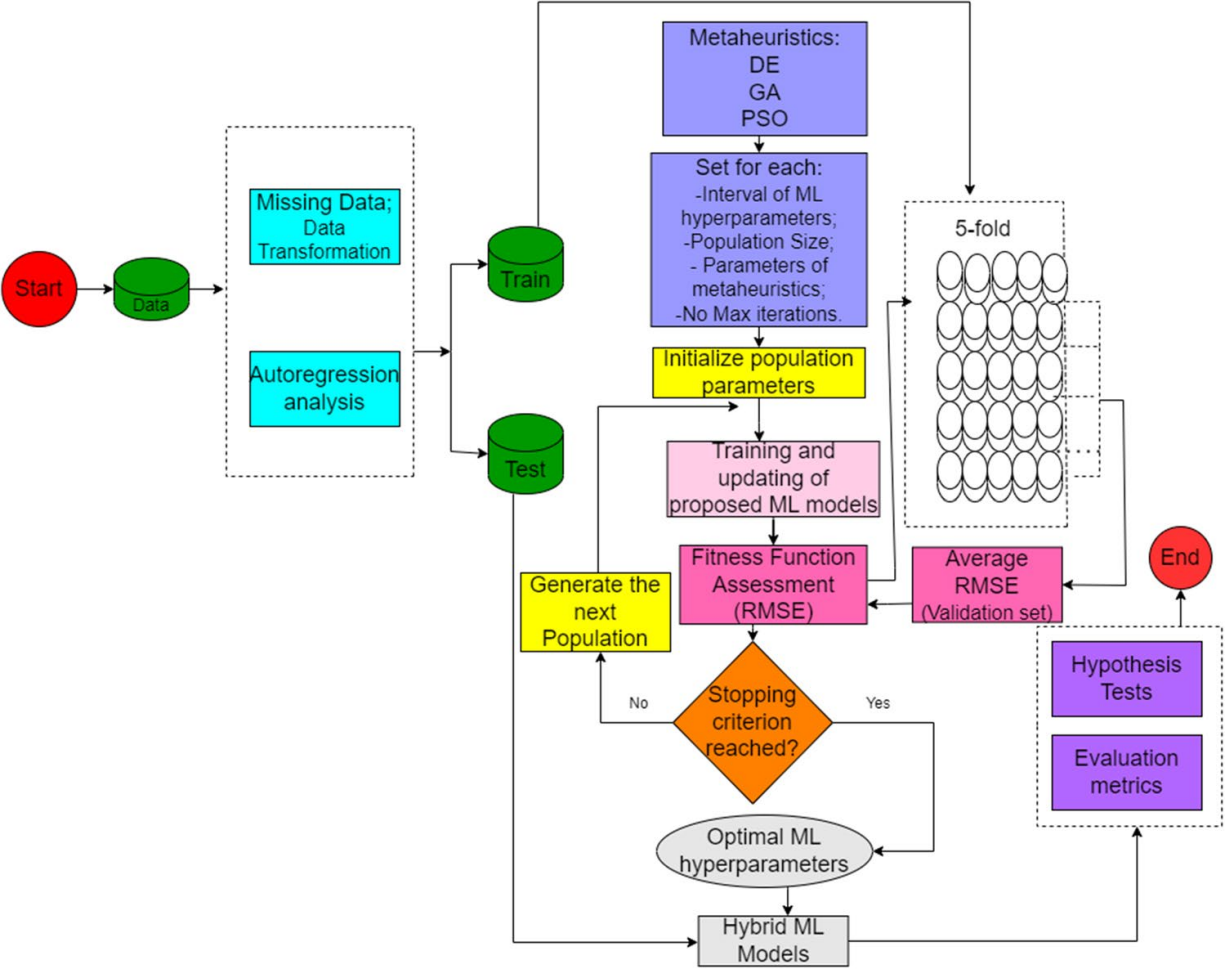
Flowchart summarizes the proposed methodology of the work.

The bioinspired algorithms, in turn, had their parameters detailed according to Table 3 (representing the classic versions of optimization algorithms). Each algorithm employed a population size of 16, consisting of randomly distributed individuals, with uniform distribution applied within the search space. Each individual is represented as a vector, comprising the hyperparameters specific to the machine learning model under analysis, and the length of this vector is determined by the number of hyperparameters relevant to each particular machine learning model. Figure 5 illustrates the flow diagram summarizing all the steps involved in the development of this work.

**Table 3:** Description of the specific parameters of the optimization algorithms.

Algorithm	Description of parameters
PSO	$\omega =0.7298$, $c_1=c_2=2.05$ (default)
GA	$cr=0.95$, $m=0.2$, crossover = ‘single’, mutation = ‘uniform’^87^
DE	$F=0.9$, $CR=0.7$, variant = 1^88^

To assess the performance of these models, eight different performance measures, which are commonly employed in hydrology to gauge the agreement between simulated and observed data^89,90^, were calculated to determine their robustness in terms of error and precision (refer to Table 4). Furthermore, statistical hypothesis tests, specifically ANOVA and Tukey tests^91^, were employed to evaluate the efficiency of the models. The distribution of performance measures, each based on 30 independent runs, was compared using the one-way ANOVA test. Subsequently, Tukey’s test was used for multiple comparisons of the performance means of the estimators (models) to identify the superior estimator among those analyzed in terms of performance, as well as for multiple comparisons of the performance means of the metaheuristics.

**Table 4:** Performance metrics used in the test set.

Metric acronym	Expression
WI	$1 - \frac{\sum _{i=1}^{N}(O_{i} - P_{i})^2}{\sum _{i=1}^{N}(\mid (P_{i} - \bar{O} ) \mid + \mid (O_{i} - \bar{O} \mid )^2}$
RMSE	$\dfrac{1}{N} \root \of {\sum _{i=1}^{N} (O_i - P_i)^2}$
MAE	$\frac{1}{N}\sum _{t=1}^{N} \mid (O_{i} - P_{i}) \mid$
MAPE	$100\times \frac{1}{N} {\sum _{i=1}^{N}}\frac{|O_i- P_i |}{|O_i |}$
NSE	$1 - \frac{\Sigma _{i=1}^{N}{(O_i - {P}_{i})}^{2}}{\sum {i=1}^{n}{(O_i - \overline{O})}^{2}}$
KGE	$1 - \sqrt{(r-1)^2 + (\alpha -1)^2 + (\beta -1)^2}$
R	$\frac{\sum _{i=1}^{N}(P_{i}-\bar{P})(O_{i}-\bar{O})}{\sqrt{\sum _{i=1}^{N}( P_{i} - \bar{P})^2 \sum _{i=1}^{N} (O_{i} - \bar{O})^2}}$

WI is the Willmott indices^92^. RMSE, MAPE, and MAE are the root mean squared error, mean absolute percentage error, and mean absolute errors, respectively. KGE is Kling–Gupta efficiency^93^. NSE (Nash–Sutcliffe efficiency)^94^. $O_i$ and $P_i$ is the real and simulated values, respectively. $\overline{O}$ is the mean of real streamflows. *r* are the Pearson’s coefficient and $\alpha$ is the proportion between simulated and real values standard deviations, and $\beta$ is the proportion between the averages of the simulated and observed values.

## Result and discussion

The flow predictions for the Cahora-Bassa reservoir were conducted with forecast horizons of 1, 3, 5, 7 days ahead, utilizing the following machine learning models: elastic net (EN), extreme learning machine (ELM), support vector regression (SVR), multivariate adaptive regression spline (MARS), and extreme gradient boosting (XGB). The parameters of these models were estimated through the use of differential evolution (DE), genetic algorithms (GA), and particle swarm optimization (PSO). This led to the development of a total of sixty (60) models, derived from the combinations of the five ML models, three metaheuristics, and four forecast horizons.

A successful execution is defined as one where the solution is both known and identified using a predetermined stopping criterion based on a maximum allowable number of evaluations. The best results among these models are denoted by being highlighted in bold.

### Performance analysis of models optimized by DE

Table 5 presents a quantitative study of the models’ performance, displaying averages and corresponding standard deviations of the performance measures for models optimized by DE across different time horizons.

The results, in general, indicate that the models achieved good performance. However, the SVR model outperformed the others in all performance measures, except for MAE and KGE, where the XGB model exhibited better results for the forecast horizon $t+1$. Conversely, for the remaining horizons ($t+3$, $t+5$, and $t+7$), XGB demonstrated superior results in almost all measures, except for MAE for $t+3$ and $t+5$, where the MARS model presented the lowest mean absolute error. It is noteworthy that while XGB did not always outperform the others, it consistently presented results very close to the best, ensuring its superiority and competitiveness in relation to the other models.

**Figure 6:**
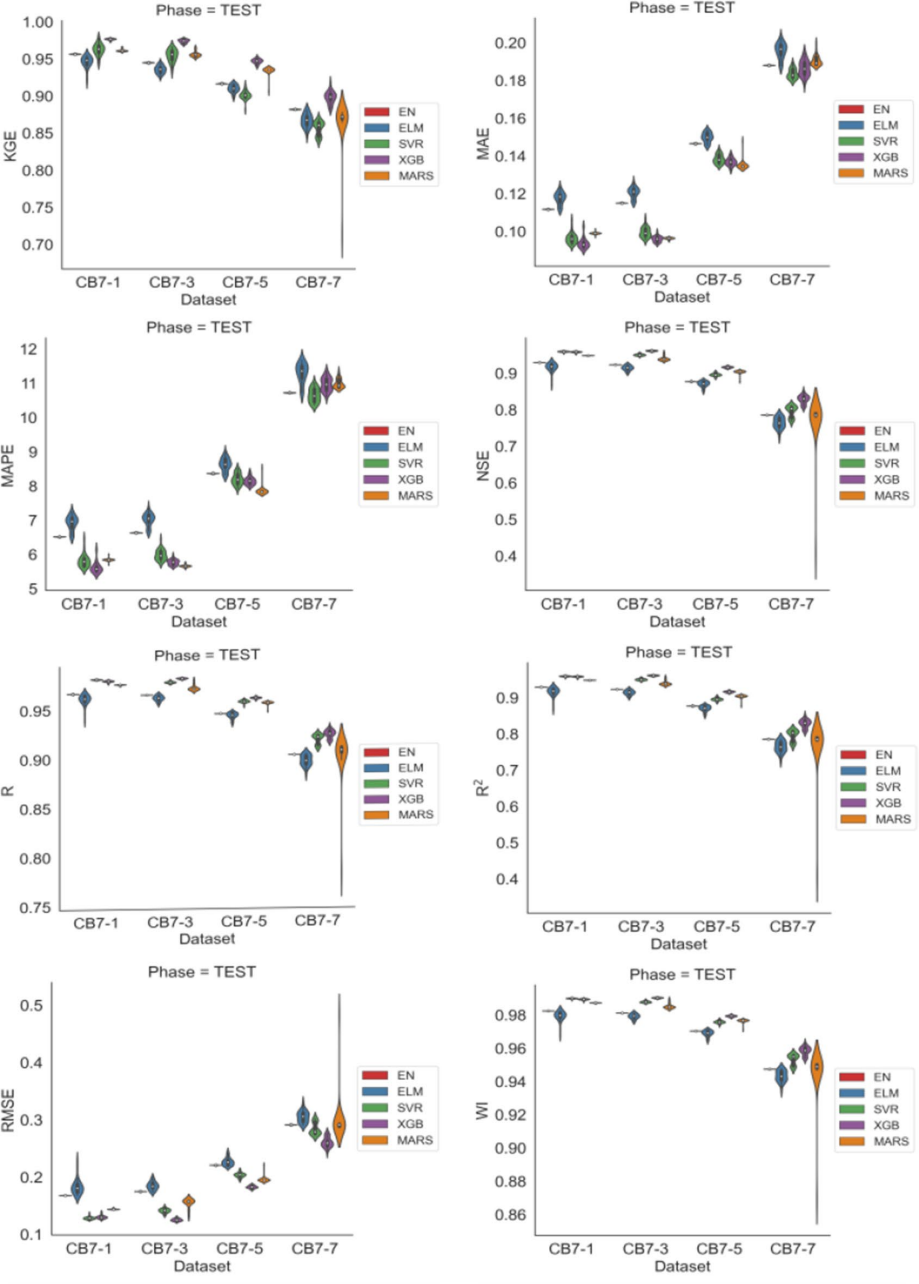
Violin to DE charts showing the distributions of the 30 runs of each metric for each model across the different forecast horizons.

Figure 6 displays violin plots representing the distributions of performance measures across different time horizons. It’s evident that these distributions exhibit positive or negative asymmetries with some influence of outliers, and MARS was the model most affected by these outliers. Furthermore, in this figure, a pattern of declining performance of the models can be observed as the time horizon increases.

**Figure 7:**
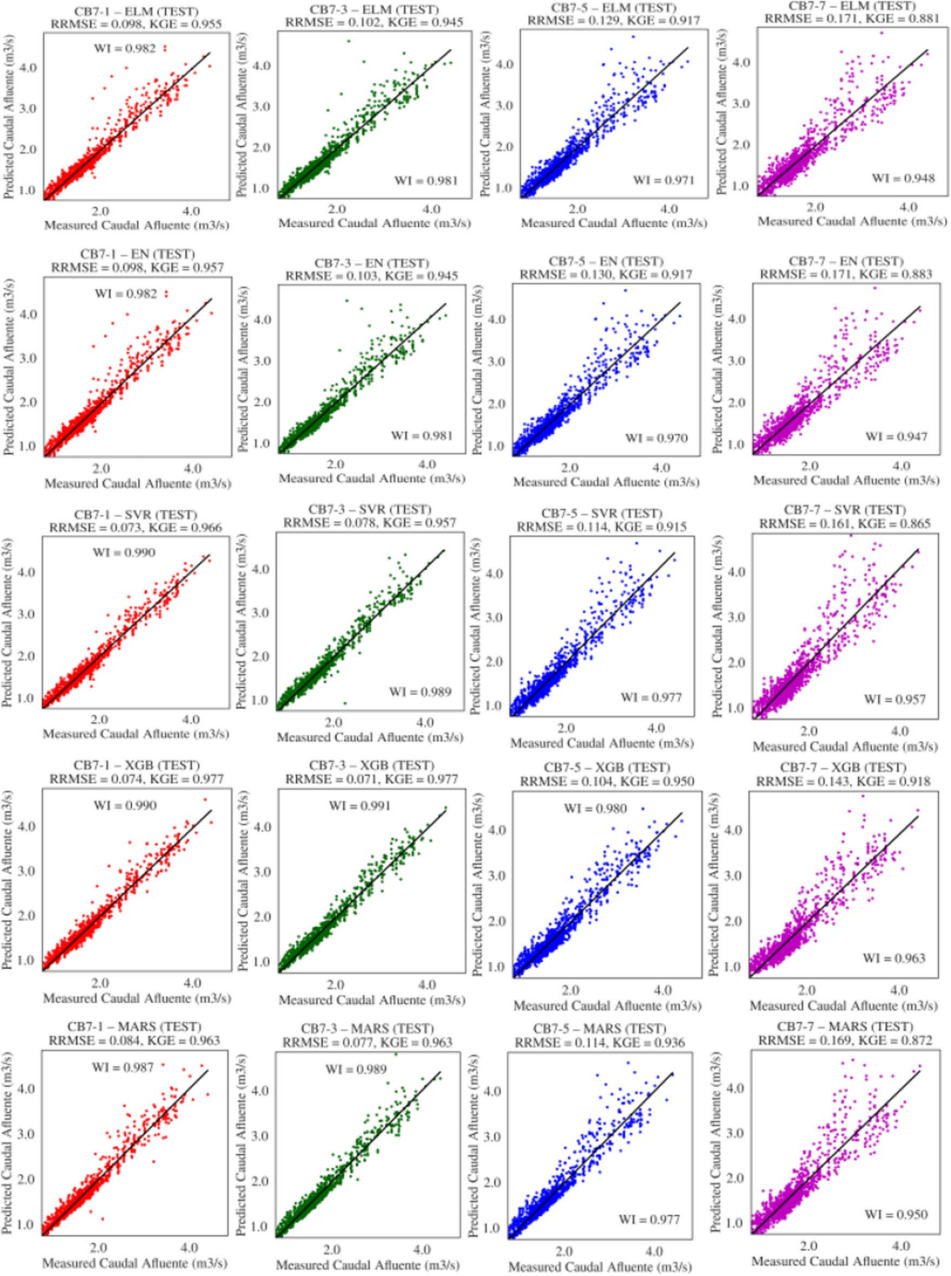
Best solution according to RMSE for flows of 1, 3, 5, 7 days ahead to DE showing levels of agreement between observed and predicted data.

Figure 7 illustrates the graphs of the best solutions for each model according to RMSE; it is generally observed that the models achieved RMSE values very close to zero, ranging between 0.071 and 0.171. Specifically, XGB had the lowest RMSE of 0.071 m$^3$/s for $t+3$, followed by SVR with 0.073 m$^3$/s for $t+1$, MARS with 0.077 for forecast $t+3$, and finally, ELM and EN both with 0.098 m$^3$/s for forecast $t+1$. Other measures of agreement between observed and predicted data, such as KGE and WI, can also be observed. The SVR model obtained values of 0.966 and 0.990 for KGE and WI, XGB achieved 0.977 and 0.991, MARS scored 0.963 and 0.989, EN obtained 0.957 and 0.982, and ELM achieved 0.955 and 0.982, respectively. Furthermore, a good approximation between observed and predicted data can be seen in these graphs, with the closest approximation achieved by the XGB model for the forecast horizon $t+3$.

**Table 5:** Descriptive statistics (means and standard deviations) of the performance measures of models optimized with DE in the test set.

DA	Estimator	R	WI	RMSE	MAE	MAPE	NSE	KGE
1	ELM	0.961 (0.006)	0.979 (0.003)	0.182 (0.013)	0.118 (0.003)	6.91 (0.210)	0.915 (0.013)	0.946 (0.008)
	EN	0.966 (0.00)	0.982 (0.00)	0.168 (0.00)	0.112 (0.00)	6.51 (0.006)	0.929 (0.00)	0.956 (0.00)
	MARS	0.975 (0.00)	0.987 (0.00)	0.144 (0.00)	0.099 (0.00)	5.84 (0.037)	0.948 (0.00)	0.961 (0.002)
	SVR	**0.981** (0.00)	** 0.990** (0.00)	**0.128** (0.002)	0.096 (0.003)	5.80 (0.182)	**0.958** (0.002)	0.963 (0.008)
	XGB	0.979 (0.00)	0.989 (0.00)	0.130 (0.003)	**0.094** (0.003)	**5.61** (0.167)	0.957 (0.002)	**0.976** (0.001)
3	ELM	0.961 (0.003)	0.979 (0.002)	0.185 (0.007)	0.121 (0.003)	7.01 (0.185)	0.913 (0.007)	0.936 (0.005)
	EN	0.965 (0.00)	0.981 (0.00)	0.175 (0.00)	0.115 (0.00)	6.63 (0.006)	0.923 (0.00)	0.945 (0.00)
	MARS	0.971 (0.003)	0.985 (0.001)	0.155 (0.008)	**0.096** (0.00)	**5.66** (0.034)	0.939 (0.006)	0.956 (0.003)
	SVR	0.977 (0.00)	0.988 (0.00)	0.141 (0.004)	0.100 (0.003)	5.98 (0.154)	0.949 (0.003)	0.954 (0.008)
	XGB	**0.981** (0.00)	**0.990** (0.00)	**0.125** (0.002)	0.096 (0.002)	5.77 (0.082)	**0.960** (0.001)	** 0.974** (0.002)
5	ELM	0.943 (0.003)	0.969 (0.002)	0.227 (0.007)	0.150 (0.003)	8.62 (0.187)	0.870 (0.008)	0.910 (0.005)
	EN	0.945 (0.00)	0.970 (0.00)	0.221 (0.00)	0.147 (0.00)	8.36 (0.005)	0.877 (0.00)	0.917 (0.00)
	MARS	0.955 (0.002)	0.976 (0.001)	0.196 (0.005)	**0.135** (0.002)	** 7.86** (0.122)	0.903 (0.005)	0.934 (0.005)
	SVR	0.957 (0.001)	0.976 (0.00)	0.203 (0.004)	0.138 (0.002)	8.20 (0.161)	0.895 (0.004)	0.901 (0.006)
	XGB	**0.960** (0.00)	**0.979** (0.00)	**0.183** (0.002)	0.137 (0.002)	8.14 (0.107)	** 0.916** (0.002)	** 0.947** (0.003)
7	ELM	0.896 (0.006)	0.943 (0.003)	0.305 (0.010)	0.196 (0.004)	11.30 (0.288)	0.764 (0.016)	0.867 (0.009)
	EN	0.902 (0.00)	0.947 (0.00)	0.291 (0.00)	0.188 (0.00)	10.72 (0.006)	0.785 (0.00)	0.882 (0.00)
	MARS	0.902 (0.023)	0.946 (0.014)	0.297 (0.035)	0.190 (0.003)	10.99 (0.132)	0.773 (0.068)	0.867 (0.029)
	SVR	0.918 (0.005)	0.953 (0.003)	0.284 (0.010)	**0.184** (0.003)	**10.65** (0.196)	0.795 (0.015)	0.856 (0.009)
	XGB	**0.923** (0.004)	**0.958** (0.003)	**0.261** (0.009)	0.187 (0.004)	10.97 (0.196)	0.828 (0.011)	** 0.898** (0.008)

### Performance analysis of models optimized by GA

Table 6 presents the descriptive statistics, average performance, and standard deviation measures produced by the forecast models whose parameters were optimized by GA.

The results indicate competitive performance among the models across all horizons: $t+1$, $t+3$, $t+5$, and $t+7$. The extreme gradient boosting (XGB) hybrid model outperforms other models for all measures and horizons, except for $t+3$ and $t+5$, where MARS resulted in the lowest MAE, and for $t+7$, where the SVR and EN models obtained the lowest MAE and MAPE, respectively. It is also worth noting that the MARS and SVR models achieved good results compared to ELM and EN.

**Figure 8:**
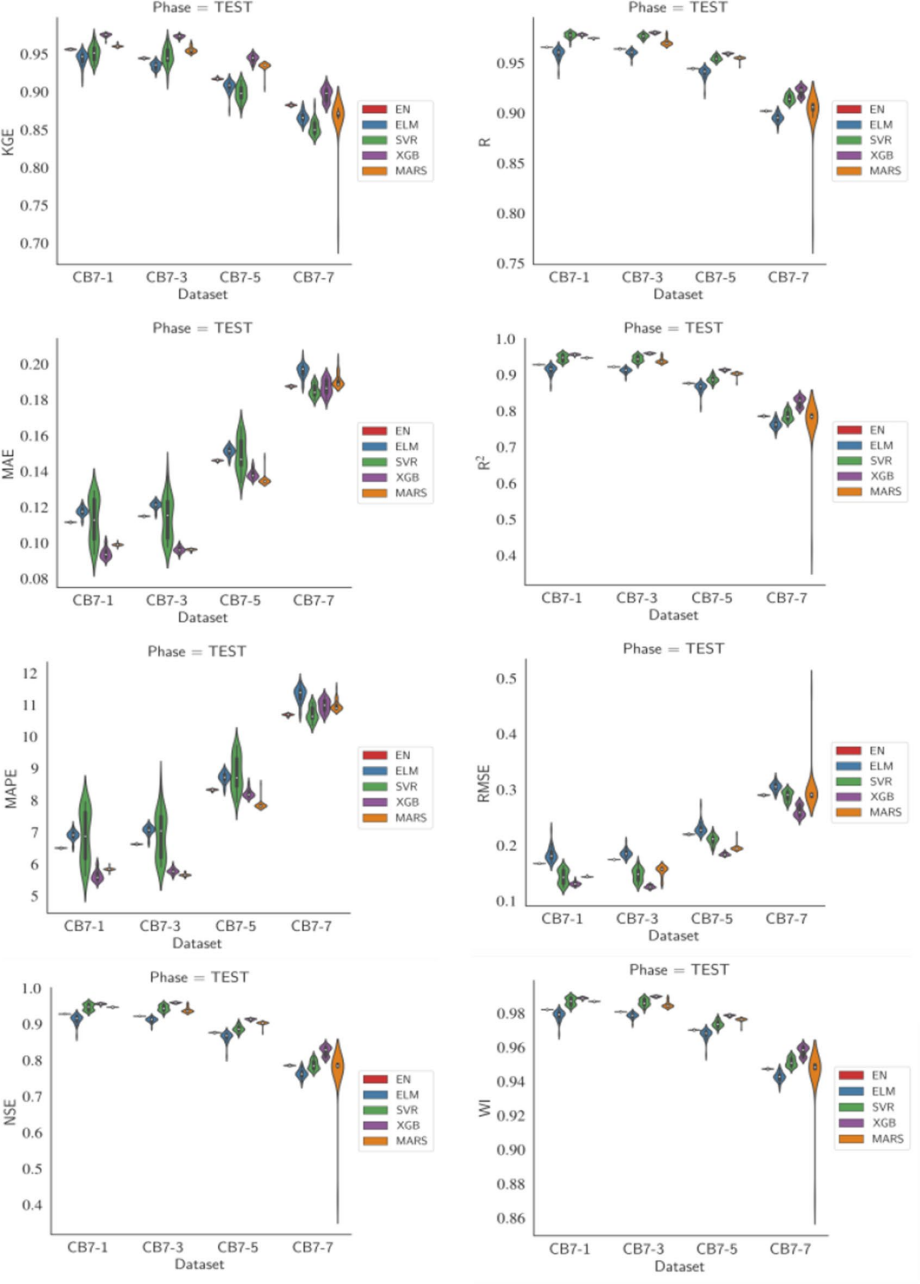
Violin plots for GA showing the distributions over 30 runs of the analyzed models on performance metrics across forecast horizons.

Figure 8 displays the distributions of the performance measures for each model across different time horizons. This figure reveals a decline in the models’ performance as the forecast horizon increases. Qualitatively, the XGB model consistently exhibits superior performance compared to the other models. Additionally, greater asymmetries are observed in the distributions of performance measures, with the presence of outliers. SVR and MARS had distributions more susceptible to extreme observations, leading to higher variability in their results.

**Figure 9:**
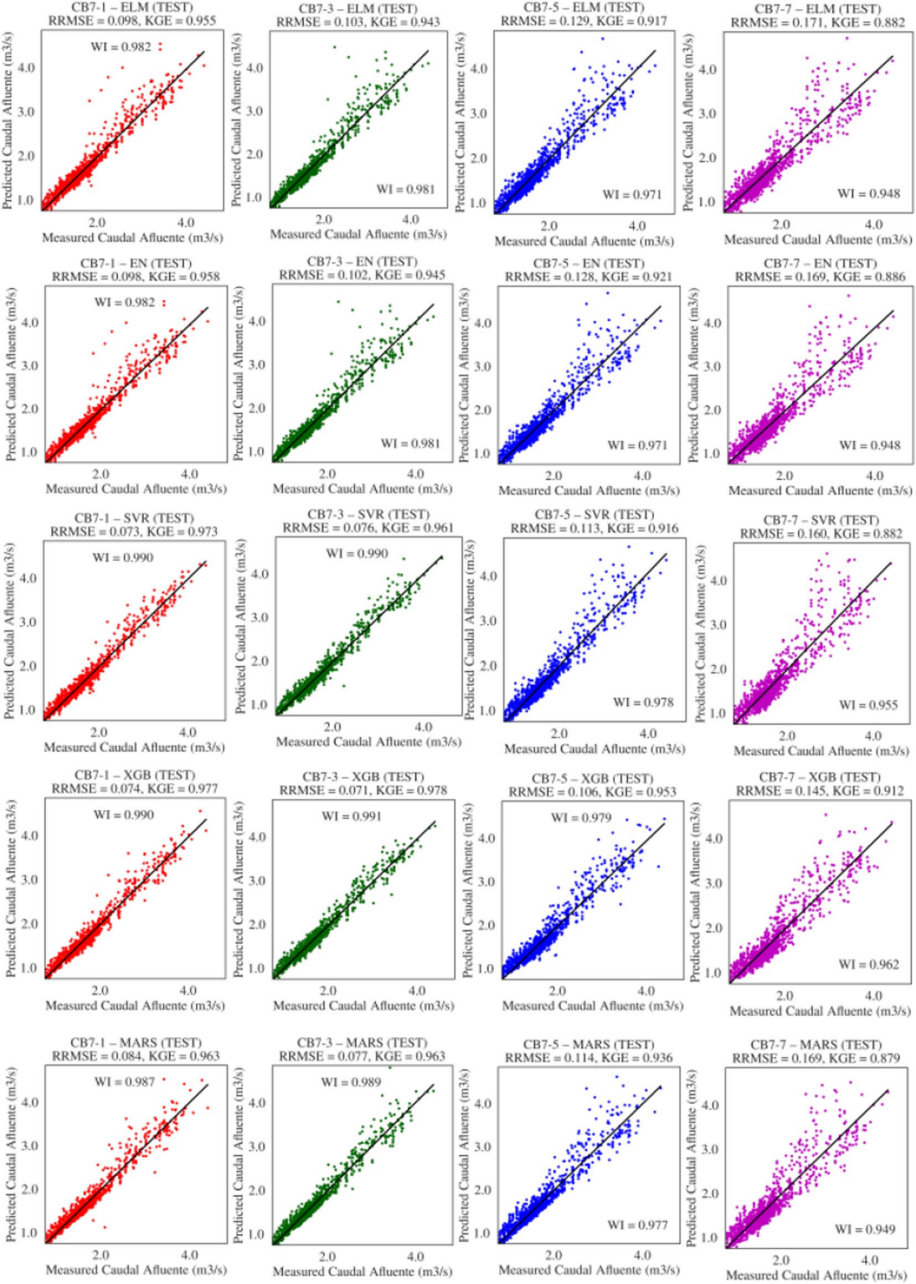
Best solution for each model according to RMSE for flows of 1, 3, 5, 7 days ahead with GA showing levels of agreement between observed and predicted data.

Figure 9 illustrates the graphs of the best solutions for each model according to the RMSE metric. Overall, the models achieved low RMSE values. However, XGB achieved the lowest RMSE of 0.071 m$^3$/s for $t+3$, followed by SVR with 0.073 m$^3$/s for $t+1$, MARS with 0.077 for forecast $t+3$, and both ELM and EN with 0.098 m$^3$/s for forecast $t+1$. Other goodness-of-fit measures can also be observed, such as KGE and WI, where the XGB model achieved values of 0.978 and 0.991, MARS scored 0.963 and 0.989, SVR obtained 0.973 and 0.990, EN achieved 0.958 and 0.982, and ELM attained 0.955 and 0.982, respectively.

Furthermore, when comparing the observed data with data predicted by the models, it can be observed that they closely align with the ideal line, indicating a good approximation between the observed and predicted data. Specifically, the XGB model with a forecast horizon of $t+3$ exhibited the best approximation.

**Table 6:** Descriptive statistics (means and standard deviations) of the performance measures of models optimized with GA in the test set.

DA	Estimator	R	WI	RMSE	MAE	MAPE	NSE	KGE
1	ELM	0.960 (0.005)	0.979 (0.003)	0.184 (0.012)	0.118 (0.002)	6.91 (0.140)	0.914 (0.012)	0.944 (0.008)
	EN	0.966 (0.00)	0.982 (0.00)	0.168 (0.00)	0.112 (0.00)	6.51 (0.008)	0.929 (0.00)	0.957 (0.00)
	MARS	0.975 (0.00)	0.987 (0.00)	0.144 (0.00)	0.099 (0.00)	5.84 (0.038)	0.948 (0.00)	0.961 (0.002)
	SVR	0.978 (0.003)	0.987 (0.002)	0.143 (0.012)	0.113 (0.012)	6.88 (0.790)	0.947 (0.009)	0.952 (0.010)
	XGB	**0.979** (0.00)	**0.989** (0.00)	**0.131** (0.003)	**0.094** (0.003)	**5.64** (0.160)	**0.956** (0.002)	** 0.976** (0.002)
3	ELM	0.961 (0.003)	0.979 (0.002)	0.186 (0.007)	0.121 (0.002)	7.06 (0.138)	0.912 (0.007)	0.935 (0.005)
	EN	0.965 (0.00)	0.981 (0.00)	0.175 (0.00)	0.115 (0.00)	6.63 (0.011)	0.923 (0.00)	0.945 (0.00)
	MARS	0.971 (0.003)	0.985 (0.002)	0.154 (0.009)	**0.096** (0.00)	**5.66** (0.039)	0.939 (0.007)	0.956 (0.004)
	SVR	0.977 (0.002)	0.987 (0.002)	0.147 (0.011)	0.114 (0.011)	6.89 (0.711)	0.945 (0.008)	0.947 (0.012)
	XGB	**0.981** (0.00)	**0.990** (0.00)	**0.126** (0.003)	0.096 (0.002)	5.78 (0.085)	**0.960** (0.002)	**0.974** (0.002)
5	ELM	0.941 (0.005)	0.968 (0.003)	0.231 (0.010)	0.151 (0.002)	8.72 (0.161)	0.865 (0.013)	0.907 (0.008)
	EN	0.945 (0.00)	0.970 (0.00)	0.220 (0.00)	0.146 (0.00)	8.33 (0.026)	0.877 (0.00)	0.918 (0.001)
	MARS	0.955 (0.002)	0.976 (0.001)	0.195 (0.005)	**0.135** (0.002)	**7.85** (0.122)	0.903 (0.005)	0.934 (0.005)
	SVR	0.955 (0.002)	0.974 (0.002)	0.210 (0.008)	0.150 (0.009)	8.85 (0.532)	0.888 (0.009)	0.899 (0.010)
	XGB	**0.960** (0.00)	**0.979** (0.00)	**0.184** (0.002)	0.138 (0.002)	8.22 (0.124)	**0.914** (0.002)	**0.945** (0.005)
7	ELM	0.896 (0.004)	0.943 (0.003)	0.306 (0.007)	0.196 (0.003)	11.32 (0.236)	0.763 (0.011)	0.867 (0.007)
	EN	0.903 (0.00)	0.948 (0.00)	0.290 (0.00)	0.188 (0.00)	**10.69** (0.032)	0.786 (0.001)	0.883 (0.001)
	MARS	0.901 (0.022)	0.946 (0.014)	0.299 (0.034)	0.191 (0.003)	10.99 (0.161)	0.771 (0.066)	0.866 (0.028)
	SVR	0.915 (0.004)	0.951 (0.003)	0.289 (0.009)	**0.185** (0.003)	10.71 (0.226)	0.787 (0.013)	0.853 (0.009)
	XGB	**0.922** (0.004)	**0.958** (0.003)	**0.262** (0.010)	0.187 (0.004)	10.98 (0.193)	**0.826** (0.013)	**0.897** (0.009)

### Performance analysis of models optimized by PSO

Table 7 presents the descriptive statistics, average performance, and standard deviation measures produced by the forecasting models with their parameters optimized by PSO.

The results demonstrated good performance of the models in all time horizons under analysis. The extreme gradient boosting (XGB) hybrid model outperformed the others in all performance measures for the horizon $t+1$. For the remaining $t+3$, $t+5$, and $t+7$, the XGB model also outperformed the other models in almost all measures, except for $t+3$ and $t+5$, where MARS presented lower MAPE and MAE results, and for $t+7$, where EN had the lowest MAPE.

**Figure 10:**
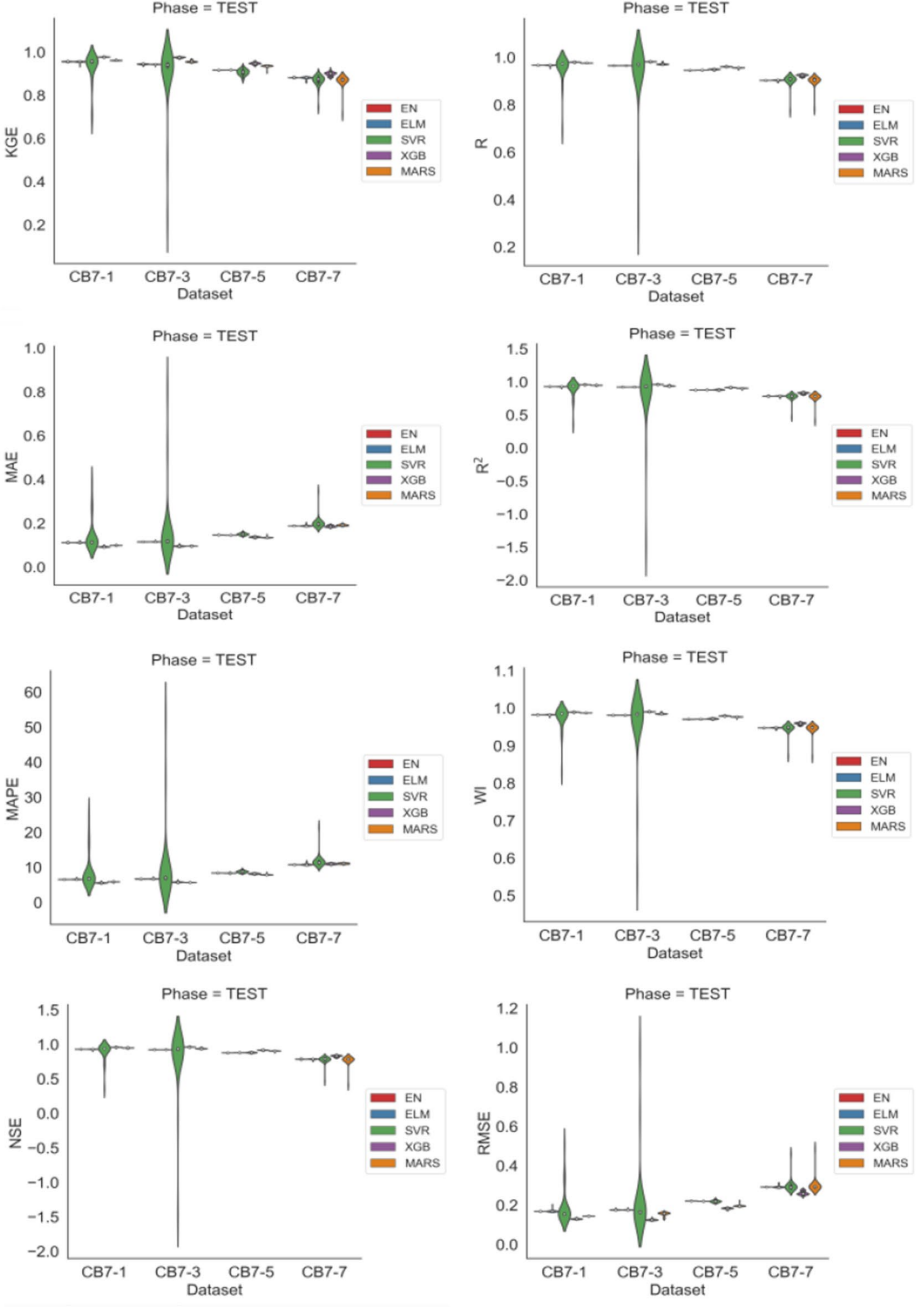
Violin plots for PSO showing the distributions over 30 runs of the analyzed models on performance metrics across forecast horizons.

Figure 10 displays the distributions of each performance measure for each model across different time horizons. The figure shows a relative balance in the performance measures of the models as the forecast horizon increases, with a downward trend. Asymmetries can also be observed in the distributions of performance measures, along with the presence of outliers. SVR exhibited the most extreme observations in its distributions, followed by MARS.

**Figure 11:**
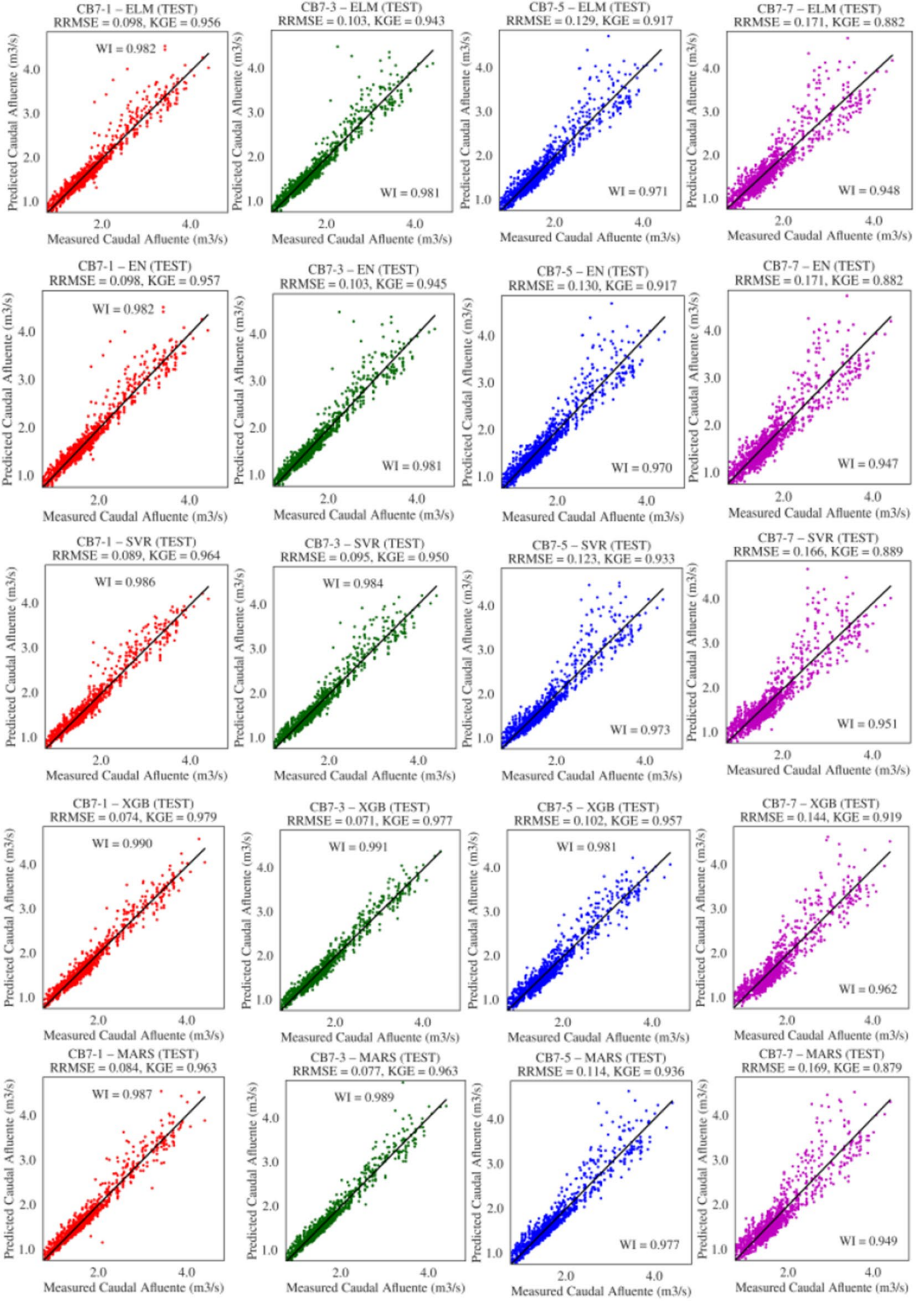
Best solution for each model according to RMSE for flows 1, 3, 5, 7 days ahead with PSO, showing levels of agreement between observed and predicted data.

Figure 11 presents the graphs of the best solutions for each model according to the RMSE metric. It is evident that the models obtained small RMSE values ranging from 0.071 to 0.171, and the XGB model achieved the smallest RMSE of 0.071 m$^3$/s. Notably, the other models also achieved relatively low RMSE values: MARS with 0.077 m$^3$/s for both $t+3$, SVR with 0.089 for forecast $t+1$, and ELM and EN both with 0.098 m$^3$/s for forecast $t+1$. Other goodness-of-fit measures can also be observed, such as KGE and WI, with the XGB model obtaining values of 0.977 and 0.991, MARS scoring 0.963 and 0.989, SVR achieving 0.964 and 0.986, EN reaching 0.957 and 0.982, and ELM attaining 0.956 and 0.982, respectively. Therefore, it can be observed that the horizons $t+1$ and $t+3$ have better fit qualities than $t+5$ and $t+7$.

Furthermore, it can be noted in this figure that there is a good approximation between observed values and those predicted by the models. When comparing the observed data and the data predicted by the models to the ideal line, it is evident that they align closely along the same line. The XGB model with forecast horizon $t+3$ demonstrated the highest level of adherence.

**Table 7:** Descriptive statistics (means and standard deviations) of the performance measures of models optimized with PSO in the test set.

DA	Estimator	R	WI	RMSE	MAE	MAPE	NSE	KGE
1	ELM	0.966 (0.002)	0.982 (0.001)	0.169 (0.006)	0.112 (0.002)	6.56 (0.116)	0.928 (0.005)	0.954 (0.004)
	EN	0.966 (0.00)	0.982 (0.00)	0.168 (0.00)	0.112 (0.00)	6.52 (0.013)	0.929 (0.00)	0.956 (0.00)
	MARS	0.975 (0.00)	0.987 (0.00)	0.144 (0.00)	0.099 (0.00)	5.83 (0.029)	0.948 (0.00)	0.961 (0.002)
	SVR	0.955 (0.055)	0.975 (0.032)	0.184 (0.081)	0.139 (0.068)	8.43 (4.52)	0.897 (0.122)	0.934 (0.066)
	XGB	**0.979** (0.00)	**0.989** (0.00)	** 0.129** (0.002)	** 0.093** (0.002)	**5.57** (0.127)	**0.958** (0.001)	**0.976** (0.001)
3	ELM	0.965 (0.00)	0.981 (0.00)	0.175 (0.002)	0.116 (0.002)	6.70 (0.112)	0.922 (0.002)	0.942 (0.002)
	EN	0.965 (0.00)	0.981 (0.00)	0.175 (0.002)	0.115 (0.00)	6.65 (0.026)	0.922 (0.002)	0.943 (0.002)
	MARS	0.971 (0.003)	0.985 (0.002)	0.155 (0.009)	**0.096** (0.00)	**5.66** (0.035)	0.939 (0.007)	0.956 (0.004)
	SVR	0.932 (0.142)	0.960 (0.090)	0.208 (0.169)	0.156 (0.143)	9.43 (9.50)	0.818 (0.460)	0.901 (0.148)
	XGB	** 0.981** (0.00)	** 0.990** (0.00)	** 0.125** (0.003)	0.096 (0.002)	5.75 (0.125)	**0.960** (0.002)	**0.974** (0.003)
5	ELM	0.946 (0.00)	0.971 (0.00)	0.219 (0.00)	0.146 (0.00)	8.32 (0.007)	0.878 (0.00)	0.917 (0.00)
	EN	0.945 (0.00)	0.970 (0.00)	0.221 (0.00)	0.147 (0.00)	8.36 (0.002)	0.876 (0.00)	0.916 (0.00)
	MARS	0.955 (0.002)	0.976 (0.001)	0.195 (0.005)	** 0.135** (0.002)	**7.85** (0.123)	0.903 (0.005)	0.934 (0.005)
	SVR	0.948 (0.002)	0.971 (0.001)	0.218 (0.006)	0.150 (0.005)	8.73 (0.316)	0.879 (0.006)	0.906 (0.014)
	XGB	** 0.960** (0.001)	** 0.979** (0.00)	**0.182** (0.003)	0.137 (0.003)	8.15 (0.126)	** 0.916** (0.003)	**0.947** (0.005)
7	ELM	0.902 (0.003)	0.947 (0.002)	0.292 (0.005)	0.189 (0.003)	10.81 (0.255)	0.783 (0.008)	0.880 (0.006)
	EN	0.902 (0.00)	0.947 (0.00)	0.291 (0.00)	0.188 (0.00)	**10.73** (0.011)	0.785 (0.00)	0.881 (0.00)
	MARS	0.901 (0.023)	0.945 (0.014)	0.299 (0.035)	0.192 (0.003)	11.05 (0.145)	0.770 (0.068)	0.867 (0.029)
	SVR	0.902 (0.025)	0.946 (0.014)	0.297 (0.031)	0.202 (0.027)	11.74 (1.85)	0.773 (0.059)	0.867 (0.028)
	XGB	** 0.923** (0.004)	**0.958** (0.003)	** 0.261** (0.009)	** 0.186** (0.004)	10.95 (0.173)	** 0.828** (0.012)	** 0.898** (0.009)

### Comparative analysis of the results and discussion

An analysis of the results obtained with different metaheuristics used in this work allowed us to quantitatively verify that, in general, all the models performed well across all the statistics used to evaluate their performance, even in relatively distant forecasting horizons. Therefore, the integration of evolutionary and/or bioinspired algorithms in the optimization of machine learning model parameters led to these positive results in multistep forecasting of daily flow.

Evolutionary/bioinspired algorithms have demonstrated their ability to find good approximations to complex problems and have achieved favorable results in determining high-quality solutions for these problems^95^, thus enabling the attainment of state-of-the-art results^59^.

Table 8 presents the results of the one-way ANOVA statistical test. The null hypothesis of the ANOVA test posits that the average on every measurement criterion is the same for all metaheuristics or estimators. As is evident, the null hypothesis is rejected for all metrics, as the p-values for each of them are less than the 0.05 significance level. This implies that all metrics are useful criteria for assessing different metaheuristics or prediction models.

**Table 8:** *p* values ANOVA test for each performance measure.

Metric	R	$\hbox {R}^2$	RMSE	MAPE	MAE	WI	NSE	KGE
*p* value	0.000	0.000	0.000	0.000	0.000	0.000	0.000	0.000

Table 9, on the other hand, presents the results of the Tukey test, which involves multiple comparisons of pairs of means from the metaheuristics. In the first column of this table, you will find the pairs of metaheuristics, followed by the mean difference in the second column, the corresponding p value in the third column, and the minimum and maximum limits of the confidence intervals associated with the differences in means for each metaheuristic pair, in the fourth and fifth columns, respectively. Lastly, the decision taken based on these comparisons is provided. The null hypothesis posits that the means of each pair of metaheuristics are equal. As observed, the null hypothesis is not rejected since all p values are greater than the significance level of 0.05. However, it’s worth noting that, despite the differences not being statistically significant, PSO exhibits relatively higher values compared to DE and GA, respectively, based on the magnitude of the p values.

**Table 9:** Test of multiple comparisons of metaheuristic means-Tukey HSD, significance = 0.05.

Pair	Meandiff	P-adj	Lower	Upper	Reject
DE-PSO	0.004	0.268	− 0.002	0.011	False
DE-SGA	− 0.001	0.900	− 0.007	0.005	False
PSO-SGA	− 0.005	0.130	− 0.012	0.001	False

PSO is a modern algorithm that has been successfully applied in engineering, demonstrating high performance compared to other metaheuristics^83,96^. It has been proven to be superior in several studies focusing on flow forecasting, with notable emphasis on the following references^26,30,35,43,44,97^, among others.

It’s important to highlight that DE and GA exhibited strongly non-significant differences. This observation aligns with the findings of Nguyen et al.^98^, who compared the performances of the extreme gradient boosting model relative to two evolutionary algorithms: genetic algorithms and differential evolution, i.e., GA-XGB and DE-XGB. Their study revealed that these models also displayed similar results.

The comparison between the machine learning models analyzed in this work is presented in Table 10, showing the results of the Tukey test ($\alpha =0.05$) for multiple comparisons of means between pairs of models. The null hypothesis assumes that the means of each pair of models are equal. The results of this test reveal the rejection of the null hypothesis, as evident from the p values of some pairs being lower than the significance level of 0.05. This indicates that the averages of certain models differ from the averages of the other models. Specifically, the extreme gradient boosting (XGB) model outperformed the other models across all metaheuristics and forecast horizons, while the elastic net model exhibited lower results.

**Table 10:** Test of multiple comparisons of means of the models-Tukey HSD, significance = 0.05.

Pair	Meandiff	p-adj	Lower	Upper	Reject
ELM-EN	0.004	0.753	− 0.005	0.012	False
ELM-MARS	0.013	0.001	0.004	0.021	True
ELM-SVR	0.008	0.068	− 0.000	0.017	False
ELM-XGB	0.030	0.001	0.021	0.038	True
EN-MARS	0.009	0.046	0.000	0.018	True
EN-SVR	0.005	0.572	− 0.004	0.013	False
EN-XGB	0.026	0.001	0.017	0.035	True
MARS-SVR	− 0.004	0.670	− 0.013	0.005	False
MARS-XGB	0.017	0.001	0.008	0.026	True
SVR-XGB	0.021	0.001	0.013	0.030	True

Table 11 illustrates all the hybrid models generated by the combination of metaheuristics and analyzed models. A total of fifteen hybrid models are obtained and compared to determine the superior model.

By combining the results analyzed separately in Tables 9 and 10, which present the Tukey tests for comparisons between metaheuristics and between models, respectively, it is evident that the extreme gradient boosting model assisted by particle swarm optimization (PSO-XGB) stands out as the superior model among all the developed models in terms of performance.

**Table 11:** Comparisons of hybrid models generated by combinations of models and metaheuristics under analysis.

ELM	(DE-ELM)	(PSO-ELM)	(SGA-ELM)
EN	(DE-EN)	(PSO-EN)	(SGA-EN)
MARS	(DE-MARS)	(PSO-MARS)	(SGA-MARS)
SVR	(DE-SVR)	(PSO-SVR)	(SGA-SVR)
XGB	(DE-XGB)	(PSO-XGB)	(SGA-XGB)
	DE	PSO	SGA

The XGB model has already demonstrated its superiority in comparison to other models when tackling machine learning challenges on various platforms such as KDD Cup and Kaggle. It has also been employed in cutting-edge applications in the industry^59^ and has been utilized for classification and regression tasks, yielding validated results in various scenarios, including customer behavior prediction, sales forecasting, hazard prediction, ad click prediction, malware rating, and web text prediction^61^.

In the context of hydrology, this model has proven to be superior to random forest (RF)^98,99^, support vector machine (SVM)^100,101^, classification and regression trees (CART)^98^, artificial neural networks^102^, and recurrent neural networks^103^ in both simple and multistep flow prediction problems. Its exceptional performance has led to its application as an alternative for flood forecasting^104^.

Models based on decision trees (or ensembles) often outperform other models, including neural networks, in regression problems.

It is interesting to note that SVR and MARS achieved competitive average results considering the evaluated metrics. However, the presence of outliers for both models had a negative impact on their performance. Despite SVR and MARS having modeling features that did not match the performance of XGB, the evolutionary search played a crucial role in finding the appropriate internal parameters that led to effective flow modeling.

The models also exhibited good qualitative adherence or approximation between the observed and estimated data, indicating that the models were capable of reproducing the characteristics of the observed data series, such as level shifts during critical periods of lower and higher flows, trends, seasonality, and other hidden characteristics with excellent quality. Therefore, these models can provide valuable support for decision-making in reservoir operations planning.

However, this performance deteriorates as the forecast horizon increases, meaning that results from shorter horizons are superior to those from relatively more distant ones. The forecast horizon introduces greater complexity to the input–output relationship involving environmental variables and flow. Additionally, longer forecast horizons amplify the uncertainty in predicting the flow’s future value. In this scenario, making accurate predictions with machine learning models becomes increasingly challenging due to the rising nonlinearity and uncertainty reflected in performance metrics.

Another observed factor that adversely affects the models’ performance is the presence of outliers, which characterize the chaotic behavior and high stochasticity of the flow^105^. As a result, there is variability in the modeled time series data, with the variation being particularly pronounced during peak flows. Model estimation of extreme events or extreme flows is challenging. However, the significance of accurately identifying extreme flows in decision-making related to dam operations is emphasized, as incorrect forecasts of these events can lead to severe consequences in water resource management.

Many models developed in the literature primarily focus on one-step or simple forecasting. Nevertheless, the results demonstrate that for one-step-ahead forecasts, it is challenging to unequivocally favor one model over the others, as the models have achieved satisfactory results. In the case of the multi-step-ahead forecasting task, the influence of the stochastic components of time series becomes more prominent with increasing forecasting time, making it difficult to identify the number of significant lags of the variable(s) that impact the prediction process^106^.

## Conclusion

In the context of sustainable and optimized water resource management and planning, the accurate prediction of flows is essential. Precise flow prediction remains a scientific challenge and has garnered significant attention due to the non-linear, non-stationary, and stochastic nature of these series.

The future of hydrological research is likely to involve maximizing information and extracting complex observations and data collected across all environmental systems to enhance the predictability of complex environmental variables. Often, predicting these variables requires extensive datasets and substantial computational resources.

This study aims to overcome these challenges by developing and evaluating five machine learning models: elastic net (EN), extreme learning machine (ELM), support vector regression (SVR), multivariate adaptive regression spline (MARS), and extreme gradient boosting (XGB). Additionally, three nature-inspired evolutionary algorithms—genetic algorithms (GA), differential evolution (DE), and particle swarm optimization (PSO)—are employed to select the internal parameters of these models. The performance of the five models is compared based on predictions at several steps (multi-steps)—1, 3, 5, and 7 days ahead of the inflow to the Cahora-Bassa dam in the Zambezi river basin, Mozambique. The data for this study were provided by the Department of Water Resources and Environment of Cahora-Bassa Hydroelectric (HCB) and cover the period from 2003 to 2018. A 5-fold walk-forward method is utilized for data partitioning into testing and training datasets.

Experiments were conducted to evaluate the forecasting capabilities of these models by applying performance measures and statistical hypothesis testing (ANOVA and Tukey). The obtained results indicate the following:

1. The nature-inspired evolutionary algorithms applied to assist in the model parameter selection of machine learning models can enhance their prediction capabilities.
2. PSO outperforms DE and GA as the superior algorithm for determining the optimal hyperparameters of ML models for forecasting, based on values obtained in each step of the considered time horizon.
3. The XGB model outperforms the others (SVR, MARS, ELM, and EN) in all evolutionary search algorithms for different forward steps, according to the performance measures and the results of the statistical tests, with the XGB model integrated with PSO being the superior model. Furthermore, SVR and MARS achieve competitive results with XGB.
4. There is good adherence or approximation of the data predicted by the models with the observed ones, even in distant horizons, indicating that the models can reproduce the characteristics of the observed data series with excellent quality. However, extreme values are predicted with some uncertainty.
5. Performance deteriorates as the forecast horizon increases, meaning that shorter horizons perform better than relatively more distant ones.

The proposed XGB hybrid model can be considered a superior alternative to the currently used models for daily flow forecasting, which is crucial for the operations of hydroelectric plants, including the allocation of the dam’s storage capacity and the optimization of operational procedures. It also plays a key role in the management of electric energy generation, the maintenance of ecological flows in the reservoir, and the continuous obtaining of flow records in non-calibrated catchments where measured flow data is unavailable.

However, it was observed that the forecasting accuracy diminishes with an increase in the forecast time. Therefore, as part of future studies, we intend to explore hybrid deep learning models, hybrid machine learning models with a multi-objective parameter selection, and variable selection techniques to analyze the reduction in the number of model inputs.

## Data Availability

Data and materials can be obtained upon request from the corresponding author (alfeudiasm@gmail.com) or Contributing author (goliatt@gmail.com).

## Code availability

Code can be obtained upon the corresponding author’s request.
